# DNMT3.1 controls trade-offs between growth, reproduction, and life span under starved conditions in *Daphnia magna*

**DOI:** 10.1038/s41598-021-86578-4

**Published:** 2021-04-01

**Authors:** Nhan Duc Nguyen, Tomoaki Matsuura, Yasuhiko Kato, Hajime Watanabe

**Affiliations:** Department of Biotechnology, Graduate School of Engineering, Osaka University, 2-1 Yamadaoka, Suita, Osaka Japan

**Keywords:** Genetics, Molecular biology, Zoology

## Abstract

The cladoceran crustacean *Daphnia* has long been a model of energy allocation studies due to its important position in the trophic cascade of freshwater ecosystems. However, the loci for controlling energy allocation between life history traits still remain unknown. Here, we report CRISPR/Cas-mediated target mutagenesis of DNA methyltransferase 3.1 (DNMT3.1) that is upregulated in response to caloric restriction in *Daphnia magna*. The resulting biallelic mutant is viable and did not show any change in growth rate, reproduction, and longevity under nutrient rich conditions. In contrast, under starved conditions, the growth rate of this DNMT3.1 mutant was increased but its reproduction was reciprocally reduced compared to the wild type when the growth and reproduction activities competed during a period from instar 4 to 8. The life span of this mutant was significantly shorter than that of the wild type. We also compared transcriptomes between DNMT3.1 mutant and wild type under nutrient-rich and starved conditions. Consistent with the DNMT3.1 mutant phenotypes, the starved condition led to changes in the transcriptomes of the mutant including differential expression of vitellogenin genes. In addition, we found upregulation of the *I am not dead yet* (*INDY*) ortholog, which has been known to shorten the life span in *Drosophila*, explaining the shorter life span of the DNMT3.1 mutant. These results establish DNMT3.1 as a key regulator for life span and energy allocation between growth and reproduction during caloric restriction. Our findings reveal how energy allocation is implemented by selective expression of a DNMT3 ortholog that is widely distributed among animals. We also infer a previously unidentified adaptation of *Daphnia* that invests more energy for reproduction than growth under starved conditions.

## Introduction

A life history trait is a major factor in determining Darwinian fitness^1–4^. Major life history traits are growth trajectories (size from the time of birth through life), age and size at maturity, age- and size-specific mortality patterns, age- and size-specific fecundity patterns^5^. Those traits are known to be negatively related to each other and these antagonistic relationships are called trade-offs^5–13^. Evolutionary ecologists have studied how an organism allocates the limited energy resources among competing traits^5, 8, 9, 14–16^. Based on energy utilization at an individual level, a theory called the Dynamic Energy budget, which can predict population dynamics, has been proposed^17^. In addition to those studies at the individual and population levels, genes affecting life history traits have been investigated in model organisms including *Drosophila melanogaster*, which led to the identification of genetic factors controlling lifespan and fecundity such as Insulin-like receptor^18, 19^, Forkhead box O (Foxo)^20^, and I am not dead yet (Indy)^21^.

The cladoceran crustacean *Daphnia magna* has long been used for studies of energy allocation. It occupies an important position in the trophic network of freshwater ecosystems, linking producers and secondary consumers. It can be easily propagated as a clone individual with a short life cycle by parthenogenetic reproduction. In addition, it can be easily manipulated both in a field and laboratory. Energy allocation of *Daphnia* fed with various amounts of algae has been investigated in order to develop a model of the interaction between the algae and *D. magna* populations^22–25^. Under caloric restriction conditions, daphnids use less energy for growth and reproduction and more for maintenance (respiration and carapace formation)^25^. Recently the *D. magna* genome has been sequenced^26, 27^ and Crispr/Cas-based genome editing technology has been established to introduce indel mutations at the targeted locus^28, 29^. However, these genetic tools have not yet been used to analyze genes involved in energy allocation.

DNA (cytosine-5) methyltransferases (DNMT; EC 2.1.1.37) are the enzymes that catalyze the DNA methylation and generate 5-methylcytosines (5mCs) to regulate gene expression^30^. There are at least 3 functional orthologs, DNMT1, DNMT3A, and DNMT3B. DNMT1 maintains DNA methylation patterns after DNA replication, while DNMT3A and DNMT3B establish new methylation by using unmethylated DNA as their templates^30^. De novo methylation by the DNMT3 family is necessary for spatio-temporal gene expression guiding cell differentiation and organogenesis in mouse embryos^31, 32^. It is also required for phenotypic changes in response to environmental stimuli^33–35^. In mice, homozygous mutants of *DNMT3A* resulted in multiple organ defects and lethality several weeks after birth^36^. Disruption of *DNMT3B* led to embryonic lethality^36^. DNMT3 orthologs harboring the mammalian DNMT3A/B-like domain structure are widely distributed in the animal kingdom^37^. In contrast to vertebrate genomes in which more than 80% of CpG dinucleotides are methylated, invertebrate genomes are sparsely methylated^38, 39^. Functional analyses of DNMT3 have been performed only in two social insects by RNA interference^33, 40, 41^, but both knockdown experiments did not lead to embryonic lethal phenotypes. However, in the honeybee (*Apis mellifera*), silencing of DNMT3 altered the developmental fate of workers-destined larvae towards queen-like traits^33^.

*D. magna* has the two DNMT3 orthologs^42^, DapmaDNMT3a.1 (*Dapma7bEVm011900t1*) and DapmaDNMT3a.2 (*Dapma7bEVm006722t1*). DapmaDNMT3a.1 lacks the PWWP domain and DapmaDNMT3a.2 has the diverged methyltransferase (MTase) domain^43^. Because both of the domains are essential for DNMT3A function, we had renamed DapmaDNMT3a.2 and DapmaDNMT3a.1 as DNMT3.1 and DNMT3.2^43^, respectively. Previously, we had found upregulation of DNMT3.1 expression in response to starvation^43^, suggesting that *DNMT3.1* may play an important role under caloric restriction*.* To further investigate the functions of DNMT3.1 under this condition, we silenced DNMT3.1 by CRISPR/Cas-mediated mutagenesis. Phenotypic analyses of this biallelic mutant demonstrated that DNMT3.1 controls life span and the trade-off between reproduction and growth only when reproduction and growth compete.

## Results

### CRISPR/Cas-mediated mutagenesis of *DNMT3.1*

**Figure 1:**
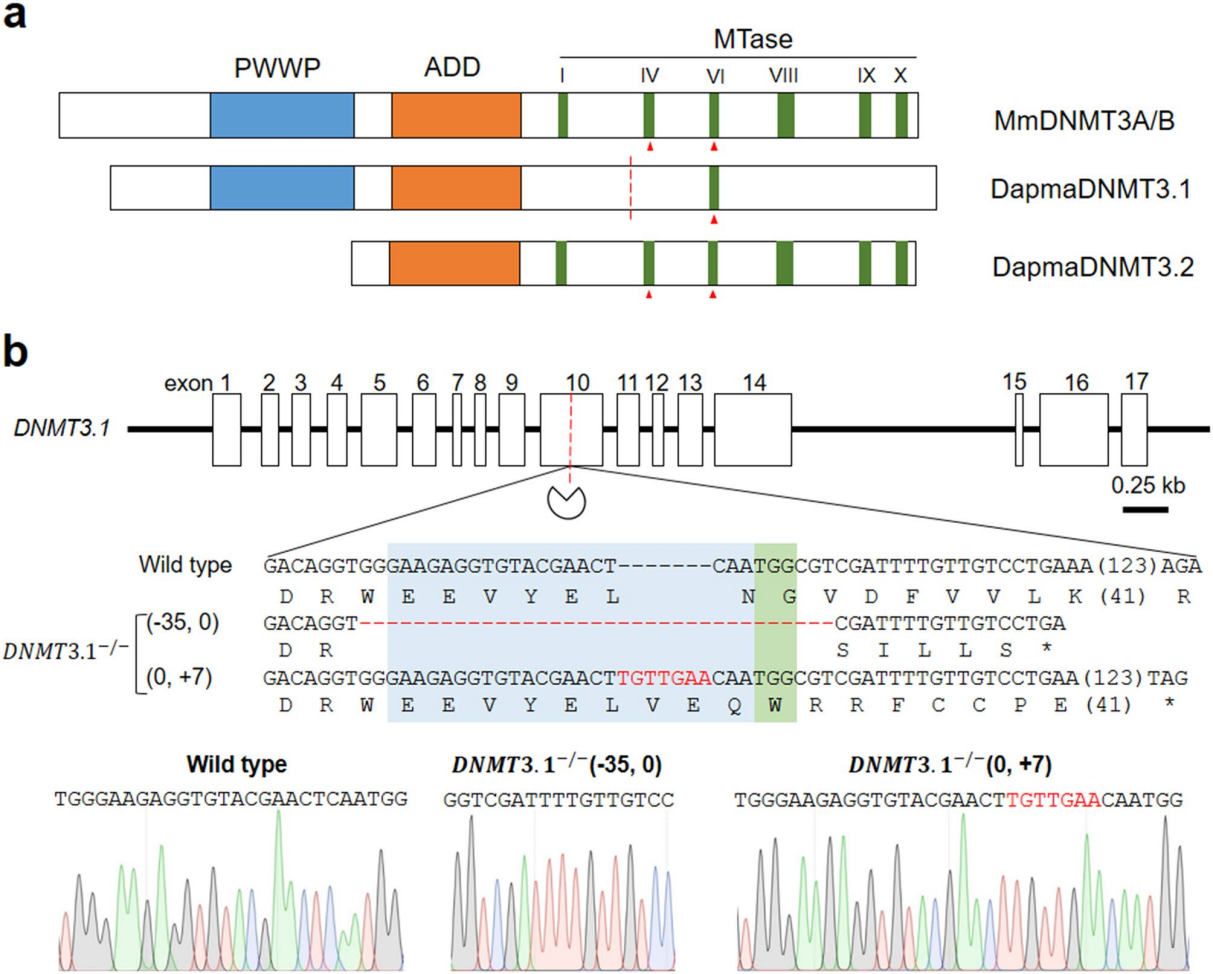
CRISPR/Cas-mediated mutagenesis of *DNMT3.1* (**a**) Schematic diagrams of domain structures of DapmaDNMT3.1 and DNM3A/B of mouse (Mm = *Mus musculus*). Red triangles indicate the active sites of catalysis. The red dashed line indicates a targeted disrupting site by CRISPR/Cas system (pie icon). (**b**) Genomic sequence of the *DapmaDNMT3.1* mutant around the gRNA-targeted site. The exons and introns of the *DapmaDNMT3.1* (*DNMT3.1*) were illustrated in white boxes and black lines, respectively. In the alignment, the top line represents the wild type *DNMT3.1* nucleotide sequence, and the deduced amino acid sequence is shown under the DNA sequence. Subsequent lines show sequences of mutant alleles followed by deduced amino acid sequences. The asterisk represents a premature STOP codon. The length (base pairs) of an abbreviated sequence and amino acid sequence are shown in parentheses among the sequences. The length (base pairs) of each indel mutation is written in the left of each sequence (−, deletions; + , insertion). The gRNA recognition and protospacer adjacent motif (PAM) sequences are in blue and green boxes, respectively. Red hyphens and letters indicate the identified mutations. Sanger Sequencing chromatograms of DNA from wild type and the biallelic mutant of *DNMT3.1* were shown below the alignment.

To investigate the functions of *DNMT3.1* in *D. manga*, we attempted to mutate this gene using the CRISPR/Cas system. As with the DNMT3L in mammals, DNMT3.1 lacks five out of six characteristic motifs of the MTase domain in catalytically active DNMT3 proteins except motif VI (Fig. 1a)^43^. gRNA was designed to bind upstream of the motif VI because the diverged MTase domain of DNMT3.1 may interact with another *D. manga* DNMT3 ortholog DNMT3.2 harboring all of the six motifs for de novo methylation as well as the interaction between DNMT3L and DNMT3A in mammals^44^. In addition, truncation of the C-terminus of DNMT3A/B led to a significant decrease of their protein levels in human embryonic stem cells^45^. Between this gRNA and the other *D. magna DNMT* genes, there are more than 6 base pair mismatches (Supplementary Fig. S2). This specificity of the gRNA to *DNMT3.1* could avoid off-target effects to the other *DNMT* genes because the DNA region with up to five base pair mismatches with gRNA is susceptible to editing by Cas9/gRNA complex^46, 47^. We injected Cas9 ribonucleoproteins (RNPs) comprising the purified Cas9 proteins and targeting gRNA into 48 parthenogenetic female eggs, of which 15 survived until the adult stage. G2 progenies of these potential founder lines were used for genotyping. We cloned and sequenced genomic PCR products encompassing the gRNA-targeted site to find mutant lines. Of the 15, we found one founder animal that produced offspring harboring biallelic indel mutations around the targeted site (Fig. 1b). The biallelic indel mutations of this line led to frameshifts, resulting in premature STOP codons occurring in both alleles (Fig. 1b). We named this mutant line *DNMT3.1*^*−/−*^ and used it for phenotyping and transcriptional analysis.

### DNMT3.1 mutation does not change growth and fecundity under nutrient rich conditions

**Figure 2:**
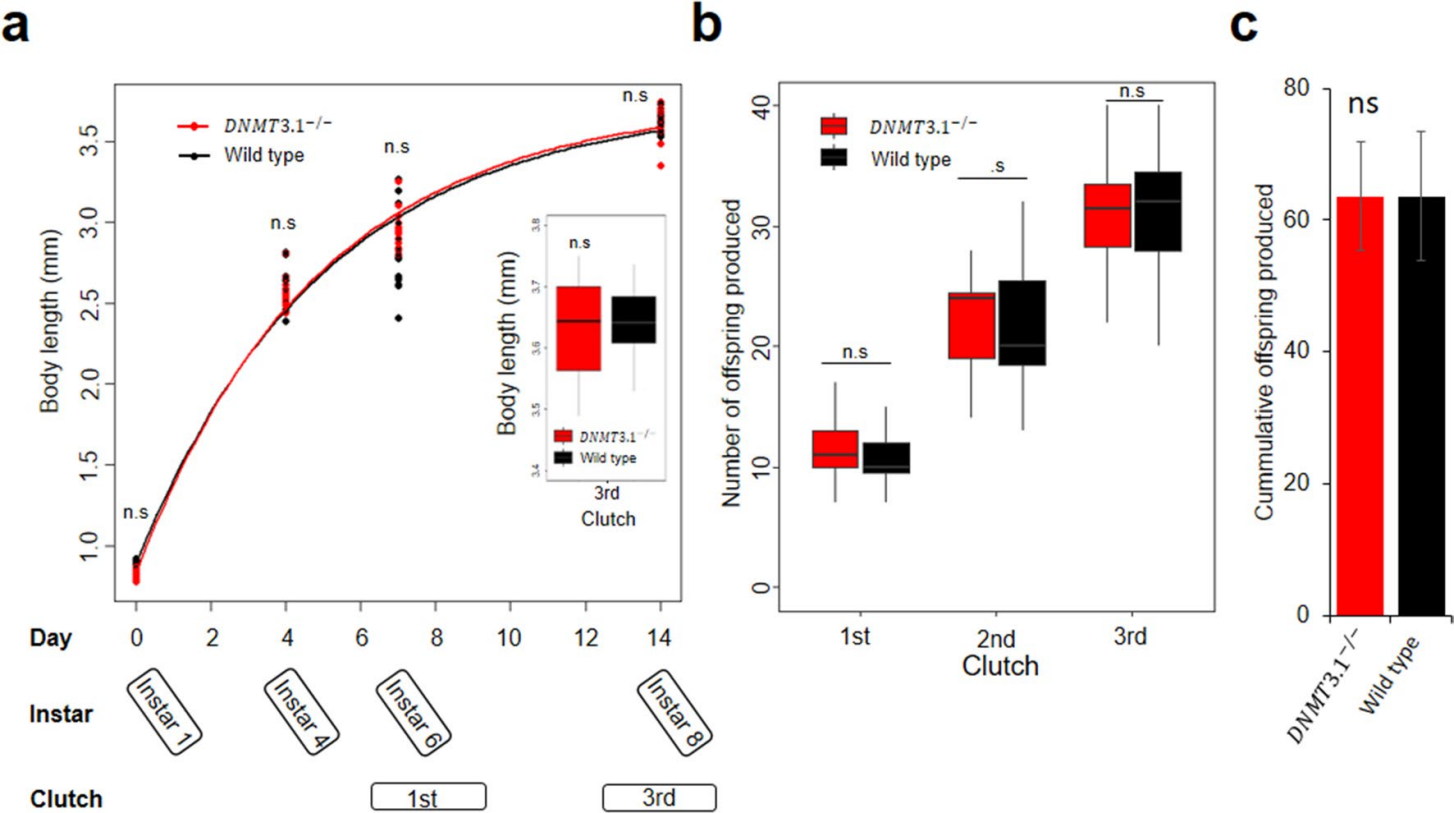
Life-history traits of wild type and *DNMT3.1*^*−/−*^ under nutrient rich conditions. (**a**) Body length measurements and fitted von Bertalanffy growth for 15 *D. magna* individuals of *DNMT3.1*^*−/−*^ and wild type. Red and black lines correspond to fitted curved for *DNMT3.1*^*−/−*^ and wild type, respectively, and boxplot of their body lengths at 3^rd^ clutch stage. (**b**) Boxplot of the 15 *D. magna*’s clutch sizes cultured individually in nutrient rich conditions. All boxplot whiskers extend to the highest and lowest values, and the boxes extended from quartile 1 to quartile 3, with the middle line showing the median. n.s. denote no significant differences by pairwise *t*-test. (**c**) Bar graph of the cumulative offspring produced from the 15 individuals at 3rd clutch. Error bars indicate the standard deviation of the mean. n.s. denote no significant difference by unpaired *t-*test.

We investigated the phenotypes of the mutant line under nutrient rich conditions (5.12 × 10^7^ *Chlorella* cells/daphnid). We cultured the wild type and *DNMT3.1*^*−/−*^ for 14 days and divided this culturing period into two phases, (1) growth phase (0–4 day) and (2) growth and reproduction phase (4–14 day) where growth and reproduction compete for energy from the food. The growth rate was measured in each phase and the number of offspring was counted in each clutch. In the growth phase, the growth rate of *DNMT3.1*^*−/−*^ was similar to that of wild type (*P* = 0.14) (Fig. 2a and Table 1). In the growth and reproduction phase, there was also no significant difference in the growth rate (*P* = 0.2) in addition to the number of offspring at each clutch or their cumulative clutch size (*P* = 0.7) between wild type and mutant lines (Fig. 2b, c and Table 1).

**Table 1:** Growth rate and clutch size of *D. magna* under nutrient rich and starved condition.

Condition	Age (day)	Instar	Phase	Growth rate		Clutch		
				Wild type	*DNMT3.1*^*−/−*^		Wild type	*DNMT3.1*^*−/−*^
Nutrient rich	0–4	1–4	Growth	0.281 ± 0.009	0.283 ± 0.007		nr	nr
	4–14	4–8	Growth + Reproduction	0.033 ± 0.005	0.035 ± 0.004	1st	10.9 ± 2.3	11.4 ± 2.9
						2nd	21.6 ± 5.5	22.3 ± 3.9
						3rd	31.1 ± 5.5	31.9 ± 5.7
Starved	0–4	1–4	Growth	0.263 ± 0.011	0.255 ± 0.02		nr	nr
	4–14	4–8	Growth + Reproduction	0.051 ± 0.01	0.082 ± 0.012***	1st	11.2 ± 1.2	10.3 ± 1.7
						2nd	13.3 ± 1.9	9.2 ± 2.6***
						3rd	8.55 ± 2.7	3.2 ± 1.7***
	14–22	8–10	Reproduction	0.007 ± 0.004	0.008 ± 0.005	4th	4.7 ± 1.5	3.85 ± 1.8
						5th	8.65 ± 2.6	8.35 ± 2.2

Values are mean ± SD; n = 15 in the nutrient rich and n = 20 in the starved. nr: not related. Significant differences between wild type versus *DNMT3.1*^*−/−*^, were indicated by asterisks via pairwise *t*-test; ***P < 0.001.

### DNMT3.1 controls trade-off between growth and fecundity under starved conditions

Because we previously had found upregulation of *DNMT3.1* expression in response to caloric restriction^43^, we compared phenotypes between wild type and *DNMT3.1*^*−/−*^ mutant under starved conditions where the number of *Chlorella* was reduced eightfold compared to that in nutrient rich conditions. We first examined the effects of starvation on growth rate and reproduction in wild type daphnids. In the growth phase, the growth rate was reduced compared to nutrient rich condition (*P* = 7.575e−06) (Supplementary Fig. S3a). In the growth and reproduction phase, the growth rate of starved daphnids was higher than that of well-fed daphnids (*P* = 1.167e−07). The starved daphnids produced a smaller number of eggs since the second clutch (Supplementary Fig. S3b, c). This reduction of the food did not lead to a significant difference in the timing of molting between nutrient rich and starved conditions.

**Figure 3:**
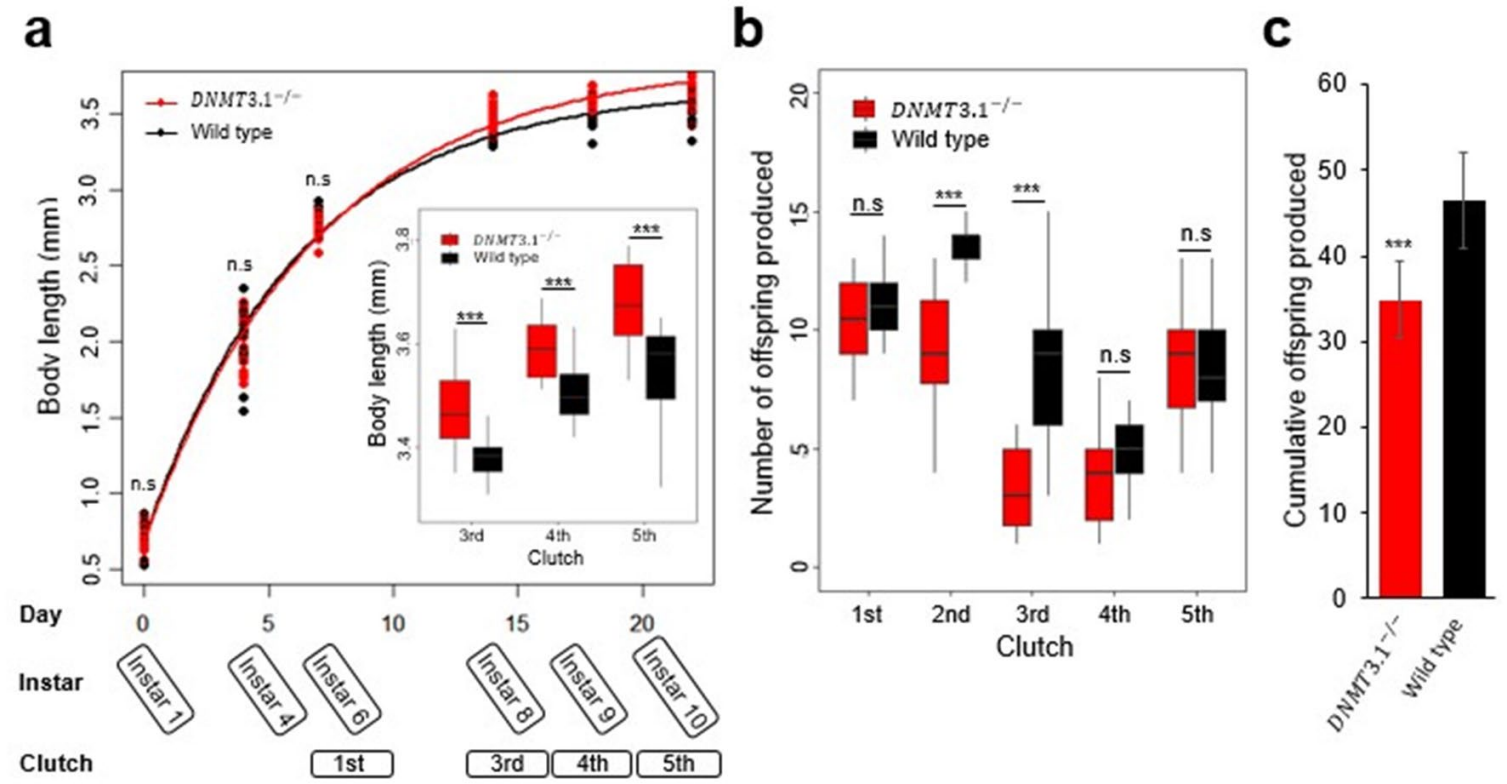
Life-history traits of wild type and *DNMT3.1*^*−/−*^ under starved condition (**a**) Body length measurements and fitted von Bertalanffy growth for 20 *D. magna* individuals of wild type and *DNMT3.1*^*−/−*^. Red and black lines correspond to fitted curves for *DNMT3.1*^*−/−*^ and wild type, respectively, and boxplot of their body lengths from 3rd to 5th clutch stages. (**b**) Boxplot of the 20 *D. magna*’s clutch sizes cultured individually in starved condition. All boxplot whiskers extend to the highest and lowest values, and the boxes extended from quartile 1 to quartile 3, with the middle line showing the median. Asterisks denote significant differences by pairwise *t*-test; n.s., not significant; ***P < 0.001. (**c**) Bar graph of the cumulative offspring produced from the 20 individuals at 5th clutch. Error bars indicate the standard deviation of the mean. Asterisks denote significant differences by unpaired *t*-test; ***P < 0.001.

We next investigated phenotypes of *DNMT3.1*^*−/−*^ mutants. There was no difference in growth (*P* = 0.12) between the wild type and the mutant in the growth phase (Fig. 3a and Table 1). However, in the growth and reproduction phase, the growth rate of mutant daphnids (0.082 mm/day) was higher than that of wild type (0.051 mm/day) (*P* = 2.5E−10) (Fig. 3a and Table 1). In contrast, mutants produced a smaller number of offspring at the second and third clutches compared to the wild type (*P* = 1.6E−06 and 8.7E−09, respectively) (Fig. 3b and Table 1). These phenotypic differences in this phase motivated us to extend observation until 22 days-old for investigations of the phenotypes. We named this third period the reproduction phase because the growth rate in this phase becomes much lower than that in the earlier two phases. In the reproduction phase, we observed no differences in growth rate and clutch size between wild types and mutants (Fig. 3 and Table 1). The timing of molting did not differ between wild type and *DNMT3.1*^*−/−*^ mutants.

### DNMT3.1 mutation reduces life span under starved conditions

**Figure 4:**
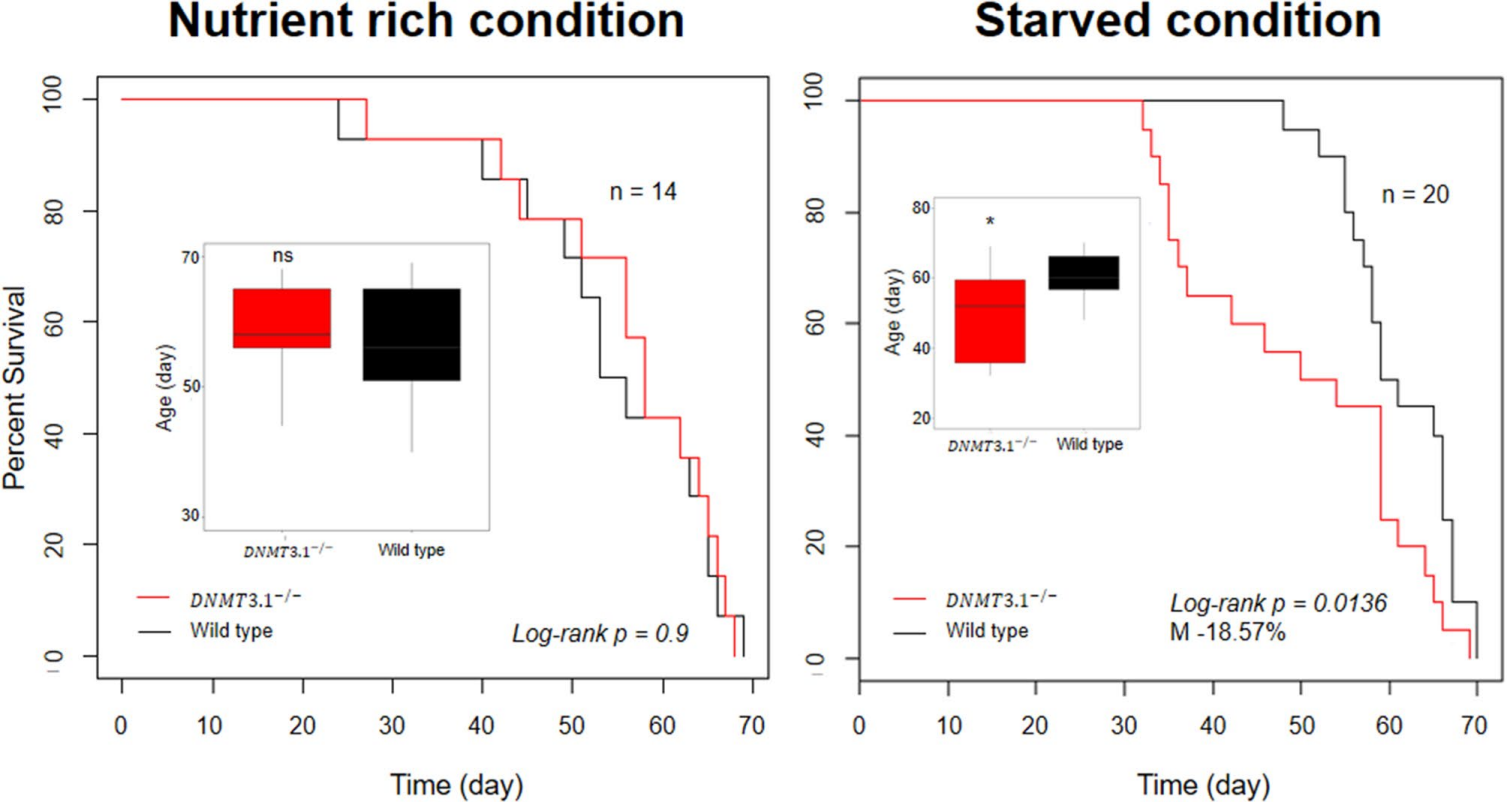
Life-span of wild type and *DNMT3.1*^*−/−*^ under nutrient rich and starved conditions. Kaplan–Meier survival curves of wild type and *DNMT3.1*^*−/−*^, and boxplot of their ages. M indicates a decrease of the median lifespan of *DNMT3.1*^*−/−*^*.* The *p* value was determined using the log-rank test. Asterisk denotes significant differences by pairwise *t*-test; n.s., not significant; *P < 0.05.

We compared the life span of the DNMT3.1 mutant to that of the wild-type. In the nutrient-rich condition, wild type and *DNMT3.1*^*−/−*^ lines showed similar longevity (log-rank *p* = 0.9), with a median lifespan of 54 and 56 days, respectively (Fig. 4). In the starved condition, a decreased trend in the lifespan of the *DNMT3.1*^*−/−*^ line was observed (log-rank *p* = 0.0136) with a median lifespan of 50 days compared to 61 days of the wild type line (Fig. 4).

### DNMT3.1 mutation alters transcriptome under starved conditions

We examined whether the other DNMT genes compensate for the DNMT3.1 mutation in this phase or not. Expression of DNMT1, DNMT3.1, and DNMT3.2 was compared by RT-qPCR between wild type and *DNMT3.1*^*−/−*^ either under nutrient rich or starved conditions. The result showed no interaction between DNMT3.1 and any of the other two DNMT genes (Supplementary Table S1). Upregulation of *DNMT3.1* expression in response to the starved condition was also confirmed both in wild type and *DNMT3.1*^*−/−*^ (Supplementary Fig. S1).

**Figure 5:**
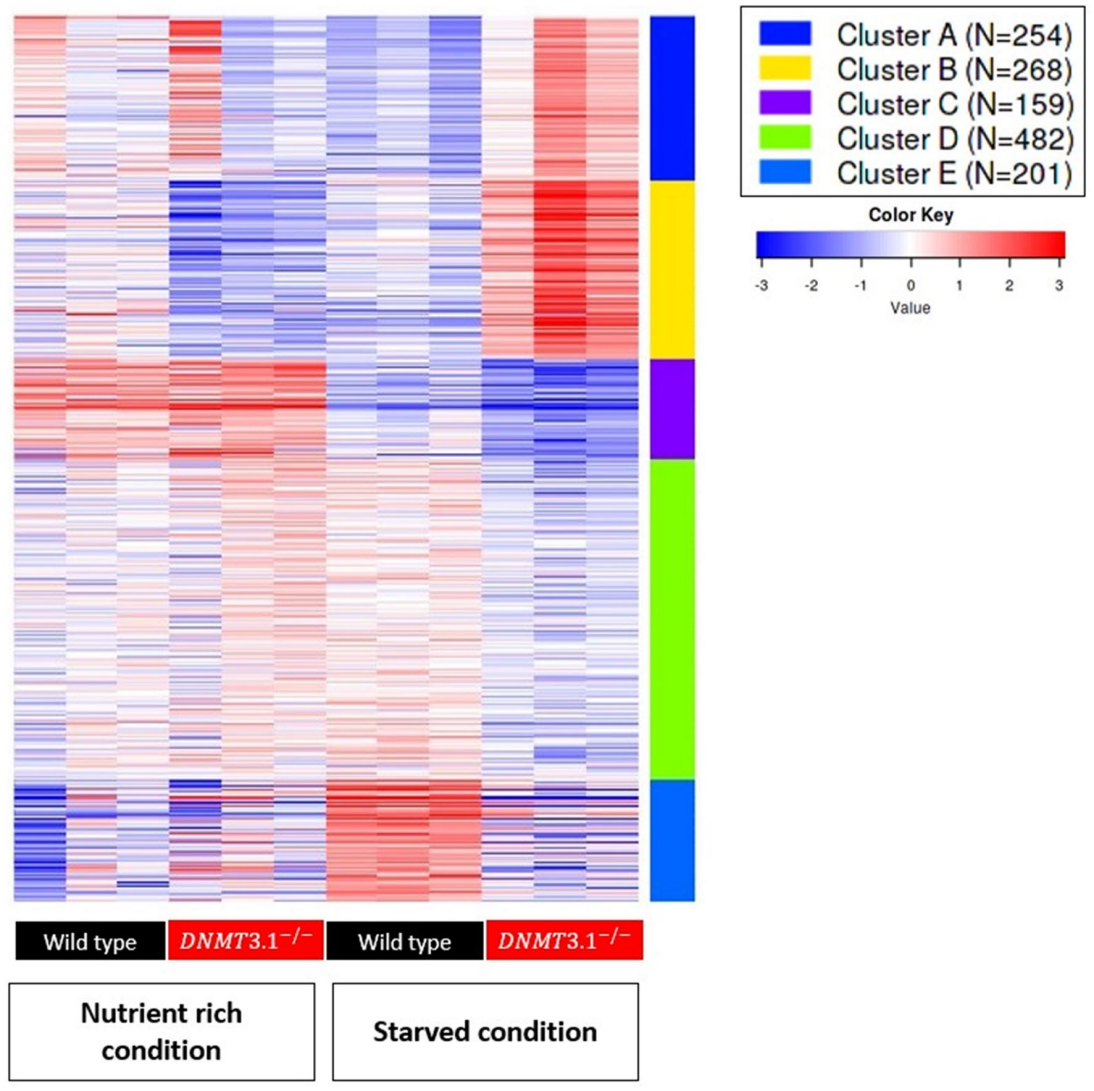
K-means clustering of DEGs from wild type and *DNMT3.1*^−/−^ in two conditions. Heatmap generated based on clustering 1364 annotated differential expression genes in the dataset into 5 clusters.

To examine how many genes were affected at mRNA level by the DNMT3.1 mutation, we performed RNA-Seq analysis to find differentially expressed genes (DEGs) between wild type and mutant lines in the second phase, growth and reproduction phase. In nutrient rich and starved conditions, numbers of differentially expressed genes were 220 and 2770 respectively, demonstrating that, in the starved condition, disruption of DNMT3.1 showed a much larger effect on the transcriptome. Amongst the DEGs in starved animals, 1432 annotated genes were identified and we performed clustering of these by k-Means. 1364 genes passed the filter (as described in Materials and Methods) and were divided into five clusters (Fig. 5, Supplementary File S1). Furthermore, we analyzed the gene ontology (GO) for functional annotation of genes in each cluster by using Fisher’s Exact Test (Supplementary File S2). Clusters A and B included genes showing higher expression in the mutant under starved conditions. The top enriched GOs in clusters A and B were mitotic cell cycle and transmembrane transport respectively (Table 2). In cluster A, we found a notable gene, *Nuclear hormone receptor ftz-f1 (Dapma7bEVm011018t1) (ftz-f1),* that showed an upregulation in the mutant under starved conditions (Table 2). Interestingly, in cluster B, we found a gene that codes for an ortholog of *solute carrier family 13 member 5* (*Dapma7bEVm010715t1*), known as *Slc13a5* or *INDY* (*I’m not dead yet*)*.* The *Daphnia INDY* ortholog showed reduced expression in the wild type but upregulated expression in the mutant under starvation (Table 2 and Supplementary File S1). Genes in Cluster C showed down-regulation in both wild type and mutant under the starved condition and were more severely down-regulated in the mutant (Fig. 5). Importantly, *vitellogenin* genes, *vtg1* (*Dapma7bEVm024402t1*), and 7 other genes related to lipid transport were included in this cluster (Supplementary File S1). Cluster D represented down-regulated genes in the mutant under starved conditions, many of which are involved in carbohydrate metabolic processes (Fig. 5 and Table 2). We found a represented gene, *target of brain insulin (Dapma7bEVm003111t1)* known as *tobi*, an α-glucosidase. Finally, genes in cluster E were upregulated in the wild type under starved conditions, which included genes related to protein turnover (proteolysis) (Fig. 5 and Table 2). A represented gene of this GO was *trypsin (Dapma7bEVm010425t1)* known as *epsilonTry*.

**Table 2:** Top gene ontology category in each cluster, and notable genes found in the cluster.

Cluster	Gene ontology (GO) term enrichment		Notable genes	Differential expression (MTvsWT)	
	Top GO biological process category	FDR *P*-value		FC	FDR *P*-value
A	Mitotic cell cycle	9.94E−17	*ftz-f1* (*Dapma7bEVm011018t1*)	2.53	4.28E-10
B	Transmembrane transport	3.83E−07	*INDY* (*Dapma7bEVm010715t1*)	41.17	0
C	Proteolysis	1.79E−13	*Vtg1* (*Dapma7bEVm024402t1*)	− 2.45	6.74E−09
D	Carbohydrate metabolic process	6.39E−17	*tobi* (*Dapma7bEVm003111t1*)	− 2.26	1.24E−05
E	Proteolysis	2.38E−09	*epsilonTry* (*Dapma7bEVm010425t1*)	− 3.22	4.22E−06

MT: *DNMT3.1*^*−/−*^ mutant*;* WT: Wild type; FDR: False discovery rate; FC: Fold change expression between mutant and wild type under starved condition.

## Discussion

Despite the long history of research on energy allocation, genes affecting energy allocation still remain largely unknown. In the cladoceran crustacean *D. magna*, energy allocation among competing life history traits under starved conditions has been extensively investigated. We have previously shown that *Daphnia DNMT3.1* was up-regulated in response to starvation, suggesting a potential function of this gene for energy allocation. In this study, we introduced a mutation into *DNMT3.1* and analyzed phenotypes of its mutant under caloric restriction. We demonstrate that this gene is a key regulator for life span and energy allocation between growth and reproduction.

### DNMT3.1 controls the trade-off between growth and reproduction under starved conditions

The trade-off between growth and reproduction occurred in both wild-type and DNMT3.1 mutants under different food levels, which is in line with previous reports^25^. Interestingly, the DNMT3.1 mutant showed a higher growth rate and lower clutch size compared to wild type when both processes competed during a period from instar 4 to 8, indicating that DNMT3.1 allocates more energy to reproduction. These results suggest that DNMT3.1 controls the trade-off between growth and reproduction when growth and fecundity compete for energy from the food. Larger clutch size has a positive impact on expanding its population size, which in turn would lead to an increase in fitness. The correlation of DNMT3.1 gene activity with phenotypes in the natural population needs to be examined to further understanding of the functions of this gene in an ecological context.

### DNMT3.1 increases life span under starved conditions

The life span of the wild type was longer than that of DNMT3.1 mutant under starved conditions. In *D. melanogaster*, starvation reduced *I am not dead yet* or *INDY* expression and in turn led to extension for longevity^48, 49^. INDY functions as a citrate transporter of the Krebs cycle and modulates energy production during the life span^50^. Interestingly, in *D. magna*, we found upregulation of *INDY* in short-lived DNMT3.1 mutants under starved conditions. Thus, we hypothesize that, in the wild type, DNMT3.1 downregulates *INDY* and extends its life span under starved conditions*.* To test this hypothesis, functional analysis of the *Daphnia INDY* ortholog would be needed in the future.

### Possible physiological function of DNMT3 ortholog in pancrustaceans

DNMT3 orthologs are phylogenetically distributed even in invertebrates but their functions still remain unclear because invertebrate models such as *Caenorhabditis elegans* and *D. melanogaster* lack the DNMT3 ortholog and DNA methylation on the genome. In *D. magna*, DNMT3.1 mutant is viable and did not show any developmental changes under nutrient-rich conditions. Consistent with this phenotype, knockdown of DNMT3 orthologs in social insects did not reduce survivability^33, 40, 41^. Although we need to confirm how much the DNMT3.1 protein level has been reduced in the DNMT3.1 mutant, in arthropods, the DNMT3 ortholog may not function in basal development, unlike mammalian DNMT3. In flies, InR and FOXO are known to control life span and fecundity^18–20^. Since a potential FOXO binding site has been found in a promoter region of DNMT3.1, core components like the FOXO signaling pathway which influence life history traits may be conserved and linked to DNMT3.1 as a downstream component. Functional analyses of DNMT3 orthologs in other pancrustaceans under stressed conditions including starvation will lead to a better understanding of the functions of this gene in the control of life history traits.

### Possible molecular function of DNMT3.1

Previously, a study in *Daphnia* has shown 115 genes with significantly hypermethylated regions under starved conditions in *D. magna*^51^. Under the starved condition, of the 115, only 8 showed reduced expression in wild type but were de-repressed upon DNMT3.1 mutation (Supplementary Table S3). This result may suggest that DNMT3.1 does not have an important role in DNA methylation under starved conditions. Because DNMT3.1 mutation changed life history traits and expression patterns of 2770 genes under starved conditions, it might regulate gene expression globally although we could not exclude the possibility that DNMT3.1 controls master regulatory factors involved in starvation responses. In mice, both DNMT3A and DNMT3B function as a transcriptional repressor without catalytic activity in cultured cells^52^ In addition, catalytically inactive DNMT3B has been demonstrated to rescue the embryonic lethal phenotype of DNMT3B knockout mice^53^, suggesting that DNMT3B has a function independent of DNA methylation. Taken together, the DNMT3.1 mutant lacking a typical MTase domain and activity has lost the control of a large amount of genes differentially expressed under the starved conditions.

Among the potential DNMT3.1 target genes under starved conditions, we listed five notable genes including *INDY* (Table 2). *ftz-f1*, a competence factor for juvenile hormone (JH) activation, has essential roles in developmental regulation and various aspects of insect adult life^54, 55^. *Vtg1* encodes vitellogenin, a precursor of a major yolk protein^56^*,* and has been used as an indicator of fecundity^42, 57^. The downregulation of *Vtg* genes was consistent with the severe decrease of fecundity in mutants in response to the starved condition. *trypsin* encodes digestive protease in the gut of *D. magna*^58^ and has been listed as involved in starvation resistance gene in flies^59^.

Our study demonstrates the physiological function of DNMT3.1 in extending longevity and energy allocation to reproduction under starved conditions. Although DNMT3.1 lacks an evolutionarily conserved sequence of the MTase domain, it changes the global transcriptome in response to starvation. In the future, the gene regulatory mechanisms can be investigated not only by investigating DNA methylation but also by chromatin immunoprecipitation for the identification of direct targets of this protein. We anticipate that this work will contribute to understanding the molecular mechanisms underlying energy allocation in the ecologically important *Daphnia* species.

## Materials and methods

### Maintenance of *D. magna* strain and the DNMT3.1 mutant

The *Daphnia magna* strain (NIES clone) was obtained from the National Institute for Environmental Studies (NIES; Tsukuba, Japan) and cultured under laboratory conditions for many generations by using ADaM as the culture medium^60^. Briefly, for maintenance of this strain, 80 individuals were cultured in the 5 l ADaM medium and daily fed with 160 μl of 7 × 10^9^ cells ml^−1^ *Chlorella* (*Chlorella vulgaris*) algae. The mutant line was maintained similarly by culturing 40 individuals in 2.5 l ADaM medium and daily feeding with 80 μl of 7 × 10^9^ cells ml^−1^ *Chlorella* (*Chlorella vulgaris*) algae. These cultures were performed at 22–24 °C, under a light/dark photoperiod of 16/8 h.

### CRISPR/Cas9-mediated mutagenesis

Impairment of the DNMT3.1 gene was done with CRISPR/Cas9 technology by introducing a frameshift and premature STOP codon as described previously^28^. For the preparation of the *DNMT3.1-*targeting Cas9 RNPs, we purified Cas9 proteins as described elsewhere^61^. For the synthesis of the gRNA, DNA templates with the T7 promoter and target site (5′-GGAAGAGGTGTACGAACTCAAtgg-3′, protospacer adjacent motif shown in lowercase), was amplified by PCR^28^ and purified by phenol/chloroform extraction. These DNA fragments were used as templates for in vitro transcription with mMessage mMachine T7 Kit (Life Technologies, California, USA), followed by column purification with miniQuick Spin RNA columns (Roche diagnostics GmbH, Mannheim, Germany), phenol/chloroform extraction, ethanol precipitation, and reconstitution in DNase/RNase-free water (Life Technologies, California, USA). We incubated 2 μM gRNA with 1 μM Cas9 protein to generate Cas9 RNPs and injected them into parthenogenetic female eggs according to established procedures^62^. Accordingly, the eggs were collected from 2–3 weeks daphnids after ovulation and placed in an ice-chilled M4 medium containing 80 mM sucrose (M4-sucrose). Approximately 0.2 nL volume of the generated Cas9 RNPs were injected by using N_2_ gas pressure through a glass needle. After injection, to investigate the Cas9-induced mutations, G2 offspring were homogenized in 90 μL of 50 mM NaOH with zirconia beads. The lysate was heated at 95 °C for 10 min and then neutralized with 10 μl of 1 mM Tris–HCl (pH 7.5). This crude DNA extract was centrifuged at 12,000 rpm for 5 min and then used as a template in genomic PCR. The targeted genomic regions in the *DNMT3.1* locus were amplified by PCR with Ex Taq Hot Start Version (Takara, Japan) and the following primers; DNMT3.1-U-gD*NA* 5′-TCCGGGTCGTGGTACTCC-3′ and DNMT3.1-D-gD*NA* 5′-AGACAAGAAACGAGCAGGTGAATAG-3′. The PCR products were analyzed by polyacrylamide gel electrophoresis and DNA sequencing.

### Culture of *D. magna* under nutrient rich and starved conditions

Before culturing under starved conditions, in order to control for maternal effects, 40 *Daphnia* of each line were cultured in 2.5 l ADaM medium and daily fed with 1.5 × 10^9^ cells ml^−1^ *Chlorella* algae for at least 3 generations. Neonates from the third clutch were randomly assigned, transferred individually to a 50 ml conical tube containing 40 ml ADaM medium, and subjected to nutrient rich or starved treatments. Under the nutrient rich condition, 5.12 × 10^7^ cells were given to each daphnid daily. For starvation, daphnids were fed with the eightfold lower amounts of the algae. During culture, the medium was changed every day in order to avoid carbon (detrital) accumulation that could differentially affect resource availability. For life-history traits observation, 15 and 20 neonates were randomly collected from culture under nutrient rich and starved conditions, respectively. Under the nutrient rich condition, the body length of daphnids was measured on days 0, 4, 7, and 14. The clutch size was recorded by counting the number of offspring produced from the 1st to 3rd clutch. During culturing, due to handling, one wild type and one mutant were killed. Therefore, we recorded the life span of 14 individuals of wild types and mutants. For starvation experiments, the body length of daphnids was measured at the two more time points, day 18 and day 22, in addition to day 0, 4, 7, and 14. Clutch size was recorded from 1st to 5th clutch. In this treatment, we could record the life span of 20 individuals both from wild types and mutants. For expression analysis by quantitative real-time PCR and RNA-seq, 5 daphnids were also cultured individually in each condition until producing 2nd clutch (day 12). Eggs were removed before homogenization for RNA extraction. Each treatment was repeated 3 times for triplicates of sampling.

### Body length and growth rate analysis

To examine the effect of starvation on *D. magna* growth, we measured body length from the center of the eye to the base of the tail^63^ using a microscope and the ImageJ software (http://rsb.info.nih.gov/ij/). The growth rates of juvenile and adult daphnids were determined by using their body length on day 0–day 4 and day 4–day 14, respectively, and calculated as

$$g= \frac{ln{L}_{1}-ln{L}_{0}}{t}$$

according to a previous study, where *L*_1_ and *L*_0_ are the final and the initial body lengths, respectively, and *t* is the time in days from the first to final observation^64^.

### RNA extraction and cDNA synthesis.

To extract total RNA, 5 female adult daphnids were collected and briefly washed. Homogenization was performed with beads using a Micro Smash machine MS-100 (TOMY; Tokyo, Japan) in the presence of Sepasol-RNA I reagent (Nacalai Tesque Inc.; Kyoto, Japan). Total RNA was isolated according to the manufacturer’s protocol, which was followed by phenol/chloroform extraction. One μg of purified total RNA was converted into the first-strand cDNA with PrimeScript II Reverse Transcriptase (Takara, Japan) and random primers (Invitrogen, Carlsbad, CA, USA) according to the manufacturer’s recommended protocol.

### Expression analysis by quantitative real-time PCR

Total RNA purification and cDNA synthesis in adult samples treated with different food levels were performed as described above. PCR was performed using a Power SYBR Green PCR Master Mix (Thermo Fisher Scientific, USA) with Mx3005P Real-Time PCR System (Agilent Technologies; CA, USA). In the presence of the appropriate primer pairs, real-time PCR amplifications were performed in triplicate at the following conditions: 2 min at 50 °C and 10 min 95 °C, followed by 40 cycles of 15 s at 95 °C and 1 min at 60 °C. Gel electrophoresis and dissociation curve analyses were performed to confirm the correct amplicon size and the absence of non-specific bands. The relative mRNA levels of juvenile samples and adult samples treated by different food levels were analyzed by normalization of the *DapmaDNMTs* transcript level to the transcript level of ribosomal protein L32^65^. The oligonucleotide sequences for qRT-PCR were used by the previous study^43^ and combined with *DapmaDNMT1* primers *(DapmaDNMT1*-forward 5′- GAAGATTCCCTTCTGCGCTATG -3′ and* DapmaDNMT1*-reverse 5′- CACGGGCTGAGAATAAGTGGT-3′).

### RNA sequencing

9 total RNA samples were extracted from triplicate samples of each of the three treatments (wild types in starved condition and mutants either nutrient rich or starved condition) were used for RNA-seq provided by Novogene service (Novogene (HK) Company Limited, based on Illumina NovaSeq platforms with paired-end 150 bp sequencing strategy (en.novogene.com). Before proceeding with the sequencing, all 9 samples passed the QC Criteria from the company and met the requirement of library construction. A summary of counted reads and percentage of total reads mapped is shown in Supplementary Table S2. In general, we obtained between 20 and 26 million reads for each sample and approximately 80% of reads could be mapped to a reference genome in *D. magna,* which is available from wFleaBase.org^66^.

### RNA-seq data analysis

Sequencing data from 9 libraries of the above samples were analyzed using CLC Genomics Workbench 12 (Qiagen) (accession number: GSE158129). The other 3 libraries of the triplicates from wild type in nutrient rich condition (accession number: GSE150821) had been prepared and sequenced elsewhere by the same methods including *Daphnia* culturing condition as in this study^67^. The *Daphnia magna* genome that is available from wFleaBase.org^66^ was used as a reference for mapping the reads. Mapping options were set at mismatch cost 2, insertion cost 3, depletion cost 3, length fraction 0.8, similarity fraction 0.8, expression value set to total counts. Differential expression analysis was performed with the ‘Transcriptomics Analysis’ toolbox, and comprised ‘experimental set-up’, where treatments pairs were analyzed with the option ‘All group pairs’. This setting uses the Wald test and reports the expression mean of each gene with a fold change between the treatment pair. Expression values were normalized using the options ‘by totals’ and ‘state numbers in read 1,000,000′. The normalized values were transformed using the “Add a Constant” set at the value ‘1′. In order to identify the differential expressed genes (DEGs) between a pair of treatments, Anova and Likelihood ratio test were performed on the transformed values for each mapped gene, and DEGs were filtered based on false discovery rate (FDR) *p*-value cutoff FDR *P* < 0.05 and fold change cutoff FC ≥ ∣1.5∣. k-Means clustering was performed by iDEP(v.9)^68^. A list of gene ID of DEGs merged counts was uploaded to iDEP and parameters for analysis were set following a previous study^69^. Accordingly, counts were filtered out by criteria of at least 0.5 counts per million in one of the samples and transformed by VST (variance stabilizing transform). Heatmap was visualized by blue-white-red color scheme and k-Means was clustered for all of the filtered genes by choosing 5 clusters. The gene ID from the *Daphnia magna* genome was annotated by NCBI homologs using Blast2GO features within OmicsBox 1.2.4^70^. The annotated genes in each cluster were examined for the enrichment of GO terms within the subsets of the assembled sequences via Fisher’s Exact Test executed within OmicsBox 1.2.4^71^.

### Statistics

Statistical analyses were conducted using statistical program R version 3.2.5^72^. Growth rate, clutch size, and median lifespan were analyzed by pairwise t-test and Student’s t-test with Welch’s correction. Fitted von Bertalanffy growth was performed by the “FSA” R package^73^. The boxplots were generated by “ggplot2” R package^74^. Kaplan Meier survival curves and log-rank test analysis were performed by the “survival” R package^75^. Two-way ANOVA and Fisher’s LSD test were performed by “agricolae” R package^76^. The level of significance was *P* < 0.05.

## Supplementary Information

Supplementary Information 1.

Supplementary Information 2.

Supplementary Information 3.
