# Supplementary material for: DNMT3.1 controls trade-offs between growth, reproduction, and life span under starved conditions in Daphnia magna
Source: Sci Rep. 2021 Apr 1;11:7326. doi: 10.1038/s41598-021-86578-4 (PMC8016896; doi:10.1038/s41598-021-86578-4)
Supplement: Supplementary file 3 — Supplementary Information 3. [file 41598_2021_86578_MOESM3_ESM.docx]

**DNMT3.1 controls trade-offs between growth, reproduction, and life span under starved conditions in *Daphnia magna***

Nhan Duc Nguyen^1^, Tomoaki Matsuura^1^, Yasuhiko Kato^1^, Hajime Watanabe^1*^

^1^Department of Biotechnology, Graduate School of Engineering, Osaka University, 2-1 Yamadaoka, Suita, Osaka, Japan

*Corresponding author

E-mail: [watanabe@bio.eng.osaka-u.ac.jp](mailto:watanabe@bio.eng.osaka-u.ac.jp)

Tel: +81-6-6879-7427, Fax: +81-6-6879-7428

# Supplementary Information


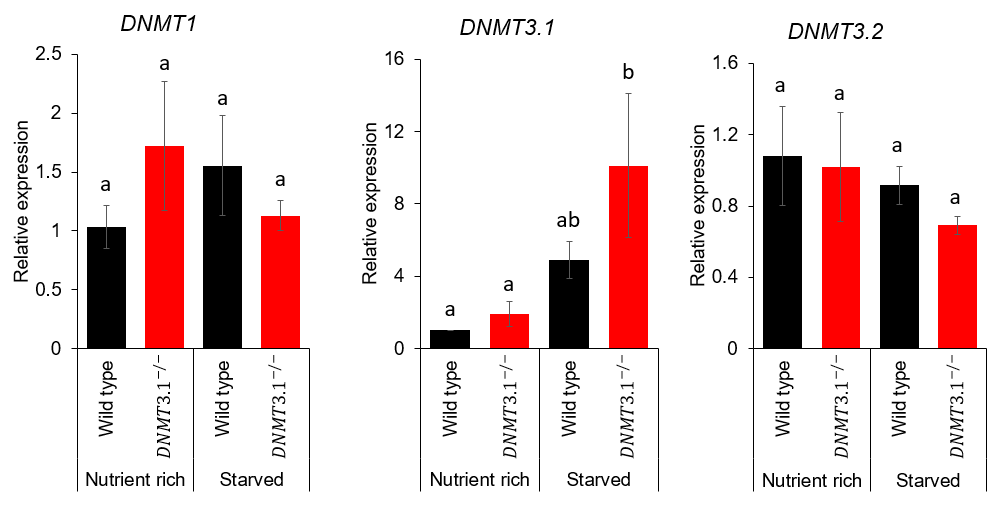


**Supplementary Figure S1.** **Relative expression of *DapmaDNMTs* in wild type and mutant under nutrient rich and starved condition** Relative expression of *DapmaDNMTs* in 12 days adults cultured in nutrient rich and starved conditions. Data were expressed as the mean fold change of starved wild type, nutrient rich and starved mutants with nutrient rich wild type as the calibrator. Normalization was performed using *Ribosomal protein L32* gene expression as the internal control. Error bars indicate the standard error of the mean (n = 3). Treatment with the different letter are significantly different (*P* < 0.05) identified by Fisher’s LSD test in multiple comparisons after two-way ANOVA.

G-GAAGAGGTGTACGAAC----TCAATGG

| | ||||| | #|| || |#||

GGCGTCGAATTGCTTTGTG--GAGGT---C-CACCTTGTC-AAGG

**a**

gRNA_DNMT3.1

*DapmaDNMT1*

gRNA_DNMT3.1 GGAAGAGGTGTACGA-ACTCAATGG

||||#|##|||| #| |#|||#|||

*DapmaDNTM3.2* AAAAAAAAATCAATATATAAGGAATATCTGTA-TATATTCA-TGG

**b**

**Supplementary Figure S2. Nucleotide sequences alignment of gRNA_DNMT3.1 against *DNMT* genes in *Daphnia magna* genome** Dash indicates gap of the alignment. Vertical bar indicates match. Hashtag indicates mismatch. The PAM site in the genomic sequences is highlighted in red text. (**a)** The alignment shows 12 gaps and 2 mismatches between the gRNA_DNMT3.1 and the genome sequence of *DNMT1 (Dapma7bEVm005001t1).* (**b**) The alignment shows 6 mismatches and 3 gaps between the gRNA_DNMT3.1 and the genome sequence of *DNMT3.2 (Dapma7bEVm011900t1).*


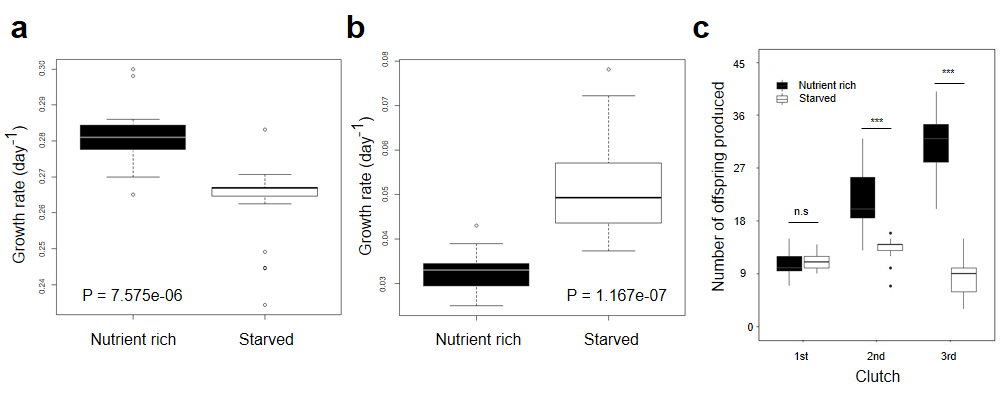


**Supplementary Figure S3. Growth rate and clutch size of wild type under nutrient rich and starved conditions** (**a**) Growth rate of wild types from day 0 to day 4 (growth phase), (**b**) Growth rate of wild types from day 4 to day 14 (growth and reproduction phase). (**a),** (**b**) Boxplot of the growth rate of 15 and 20 wild types cultured individually in nutrient rich and starved conditions, respectively. All boxplot whiskers extend to the highest and lowest values, and the boxes extended from quartile 1 to quartile 3, with the middle line showing the median. Significance was analyzed using the student’s t-test with Welch’s correction, P<0.05. (**c**) Boxplot of the clutch sizes of 15 and 20 wild types cultured individually in nutrient rich and starved conditions. Asterisks denote significant differences by student’s t-test with Welch’s correction; n.s., not significant; ****P* < 0.001. The figure was generated and statistically analyzed by R software ^1^ and ggplot2 package ^2^.

**Supplementary Table S1. Summary of two-way ANOVA for food level and line on the expression of *DNMT* genes.** The table was generated by R software ^1^ and agricolae package ^3^.

| **Gene** | **Source of variation** | **Df** | **SS** | **MS** | ***F*** | ***P*** |
| --- | --- | --- | --- | --- | --- | --- |
| *DNMT1* | FOOD (F) | 1 | 0.0037 | 0.00367 | 0.0092 | 0.9261 |
|  | LINE (L) | 1 | 0.0547 | 0.05467 | 0.1363 | 0.7216 |
|  | Interaction F × L | 1 | 0.9241 | 0.92408 | 2.3031 | 0.1676 |
| *DNMT3.1* | FOOD (F) | 1 | 109.324 | 109.324 | 8.3404 | **0.0202** |
|  | LINE (L) | 1 | 28.275 | 28.275 | 2.1571 | 0.1801 |
|  | Interaction F × L | 1 | 13.954 | 13.954 | 1.0645 | 0.3323 |
| *DNMT3.2* | FOOD (F) | 1 | 0.18253 | 0.182533 | 1.3241 | 0.2831 |
|  | LINE (L) | 1 | 0.06163 | 0.061633 | 0.4471 | 0.5225 |
|  | Interaction F × L | 1 | 0.0192 | 0.0192 | 0.1393 | 0.7187 |

Df, Degrees of freedom; SS, Single squares; MS, Mean squares. Significant effect is highlighted in bold (*P* ≤ 0.05)

**Supplementary Table S2. Summary of RNA-Seq reads**

| **Line** | **Condition** | **Replicate** | **Number of reads** | **% of total mapped** |
| --- | --- | --- | --- | --- |
| Wild type | Starved | 1 | 20,708,586 | 82.85 |
|  |  | 2 | 21,726,268 | 80.03 |
|  |  | 3 | 22,470,222 | 83.00 |
| *DNMT3.1^-/-^* | Nutrient rich | 1 | 24,195,222 | 80.14 |
|  |  | 2 | 25,194,166 | 80.48 |
|  |  | 3 | 23,052,612 | 80.93 |
| *DNMT3.1^-/-^* | Starved | 1 | 21,132,788 | 82.33 |
|  |  | 2 | 26,078,976 | 82.14 |
|  |  | 3 | 22,874,240 | 82.66 |

**Supplementary Table S3.** **Gene expression of a hypermethylated gene from wild type (WT) and mutant (MT) in starved conditions**

| **Gene ID** | **Gene name** | **DMR** | **WT** | **MT** |
| --- | --- | --- | --- | --- |
| Dapma7bEVm004172t1 | SHC SH2 domain-binding protein | Hypermethylated | Down | No |
| Dapma7bEVm010768t1 | Surfeit locus protein | Hypermethylated | Down | No |
| Dapma7bEVm003001t1 | Structural maintenance of chromosome 2 1 protein | Hypermethylated | Down | No |
| Dapma7bEVm005181t1 | conserved protein | Hypermethylated | Down | No |
| Dapma7bEVm004398t1 | Upstream stimulatory factor | Hypermethylated | Down | No |
| Dapma7bEVm000396t1 | Peroxisomal targeting signal 1 receptor | Hypermethylated | Down | No |
| Dapma7bEVm002983t1 | GTP-binding nuclear protein Ran | Hypermethylated | Down | No |
| Dapma7bEVm001440t1 | Eukaryotic peptide chain release factor gtp-binding subunit erf2 translation release factor 3 erf3 erf-3 | Hypermethylated | Down | No |

DMR: Differential methylated region reported in (58), No: no change; Down: Down-regulated; Up: Up-regulated

# References

1. RCore Team. R: A language and environment for statistical computing. (2016).
2. H. Wickham. ggplot2: Elegant Graphics for Data Analysis. Springer-Verlag New York (2016).
3. Filipe de Mendiburu. agricolae: Statistical Procedures for Agricultrual Research. *R package version 1.3-3.*( 2020). https://CRAN.R-project.org/package=agricolae
